# Oral health effects of botulinum toxin treatment for drooling: a systematic review

**DOI:** 10.4317/medoral.24101

**Published:** 2020-12-19

**Authors:** Luisa Barreto Costa Corrêa, Maurício Bartelle Basso, Bernardo Sousa-Pinto, Soraya Coelho Leal

**Affiliations:** 1 DDS, MSc. MEDCIDS. Department of Community Medicine, Information and Decision Sciences, Faculty of Medicine, University of Porto, Portugal; 2 State Health Secretariat of Federal District, Brasília, Distrito Federal, Brazil; 3 DDS, MSc. State Health Secretariat of Federal District, Brasília, Distrito Federal, Brazil; 4 MD, PhD. CINTESIS - Center for Health Technology and Services Research, University of Porto, Portugal; 5 DDS, PhD. Faculty of Health Science, Department of Dentistry, University of Brasilia, Brazil

## Abstract

**Background:**

Drooling is a major morbidity in several neurological diseases. Intraglandular botulinum neurotoxin (BoNT) injections have been used to manage this condition. However, by decreasing salivary flow, BoNT injections may result in an increased risk of caries and other oral adverse effects. In this study, we aimed to assess whether, in patients with drooling, intraglandular BoNT injections are associated with increased dental caries development, modifications on salivary composition (oral pH, buffering capacity and osmolality) and cariogenic bacterial load.

**Material and Methods:**

We performed a systematic review, searching PubMed, CENTRAL, Web of Science, and Scopus for all experimental and observational studies reporting on adverse effects of intraglandular BoNT injections in patients with drooling. Primary study selection, quality assessment, and data extraction were independently performed by two researchers. No studies were excluded based on their language, publication status or date of publication. Studies’ quality was based on revised Cochrane Risk of Bias tools. Meta-analysis was not performed.

**Results:**

We retrieved 1025 studies, of which 5 were included. Two studies were two randomized controlled trials and three quasi-experimental studies. None of the included studies found BoNT injections to be associated with dental caries development or with significant reductions in oral pH. One of the included primary studies even observed an increase in salivary buffer capacity. One study found an increase in Lactobacilli counts. As for the risk of bias, two studies were classified as having a critical risk, two as high risk and one as having some concerns.

**Conclusions:**

Currently, there is no evidence that, in patients with drooling, BoNT injections associate with increased risk of dental caries or disturbances in oral pH or salivary buffering capacity. However, the included primary studies had important limitations and differences in their methodologies.

** Key words:**Neurological diseases, drooling, sialorrhea, botulinum toxin, oral health, caries, saliva.

## Introduction

Drooling is a major morbidity in several neurological diseases (1). It usually results in maceration and infections of the peri-oral skin (1-4), dysfunctional eating (4), disturbed speech (1,2,4,5), halitosis (4), aspiration-related pulmonary complications (1-5), and stigmatization or social isolation (1,2). Also, drooling often poses hygienic problems for caregivers because of constant soiling of clothes (1) and other objects (4). This prompts the need for effective and safe approaches to manage drooling (3). One therapeutic approach consists of injecting botulinum toxin (BoNT) into the salivary glands (2,3,6). The literature (2,7,8) has shown that BoNT injections are effective in the management of sialorrhea, with most patients reporting a transient improvement in their symptoms (9,10).

Botulinum toxin acts by transiently blocking parasympathetic and postganglionic sympathetic acetylcholine release (11-13). In general, parasympathetic stimuli increase the output of water and electrolytes, whereas, when sympathetic stimuli dominate, there is an enhancement of protein synthesis and secretion from acinar cells (14,15). Therefore, by interfering with autonomic innervation of salivary glands and, thus, prompting a decrease in salivary flow, BoNT can affect the defense mechanism of saliva and may be associated with changes in the salivary composition (16,17) and increased risk of dental caries development (1,2). However, evidence regarding intraglandular BoNT adverse events in oral health has not yet been systematically assessed.

Therefore, the objective of this study is to perform a systematic review of experimental and observational studies in order to assess whether, in patients with drooling, BoNT injections into the salivary glands associate with increased risk of dental caries development, modifications on salivary composition such as salivary pH value, buffering capacity of saliva and osmolality - and modifications on counts of cariogenic bacteria, including salivary counts of *Streptococcus mutans* (S.mutans) and *Lactobacilli*. In addition, this study aimed to assess the methodological quality of existing evidence on the safety of intraglandular BoNT for treatment of drooling, discuss the main limitations of the current evidence, as well as to produce methodological recommendations for future studies on this field.

## Material and Methods

This systematic review was conducted in accordance with the "Preferred Reporting Items for Systematic Reviews and Meta-Analysis (PRISMA) statement (18) and has been registered on PROSPERO database (Registration CRD42019137023).

We included experimental and observational studies in which BoNT injections into salivary glands were used to treat drooling irrespective of patients’ underlying disease. We included studies that reported on adverse events of BoNT on oral health, particularly regarding (i) caries development; (ii) modifications of salivary composition (salivary pH value, buffering capacity of saliva, and hydration level - osmolality); and (iii) modification of salivary counts of cariogenic bacteria, especially *Streptococcus mutans* and *Lactobacilli*. No studies were excluded based on their language, publication status or date of publication.

We searched PubMed, CENTRAL, Web of Science and Scopus in May 2019 to identify relevant primary studies. Manual searching was also performed to collect data reported in books and conference abstracts, as well as by searching references of included primary studies. The search queries can be found on Supplement1 (http://www.medicinaoral.com/medoralfree01/aop/24101_Supplement1.pdf). After removing duplicates, two reviewers (LC and MB) were independently involved in selecting the studies, firstly by title and abstract reading, and then by full-text reading.

Data were independently extracted from primary studies by two researchers, using specifically designed forms. Extracted data from primary studies included the number of patients and their sex and age distribution, underlying/previous diseases, BoNT type, administration dosage of BoNT, number of botulinum toxin sessions, administration dosage of BoNT, site of application of BoNT, follow-up time, salivary pH value, buffering capacity of saliva, osmolality, and salivary counts of the cariogenic bacteria (especially *S. mutans* and *Lactobacilli*). Authors of the included primary studies were contacted for providing eventual missing data. The quality of primary studies was independently assessed by two authors based on the Revised Cochrane risk-of-bias tool for randomized trials (19) (RoB 2.0 tool) and Risk of Bias in Non-randomized Studies - Interventions (ROBINS-I) (20) questionnaire for non-randomized studies. Any disagreement was solved by discussing with a third researcher.

Results were presented using descriptive statistics. We performed the Chi-square test to assess the statistical significance of the incidence of carious lesions in one of the included primary studies (2). Meta-analysis was not performed due to the small number of included studies and the relevant methodological and clinical differences between them.

## Results

**Figure 1:**
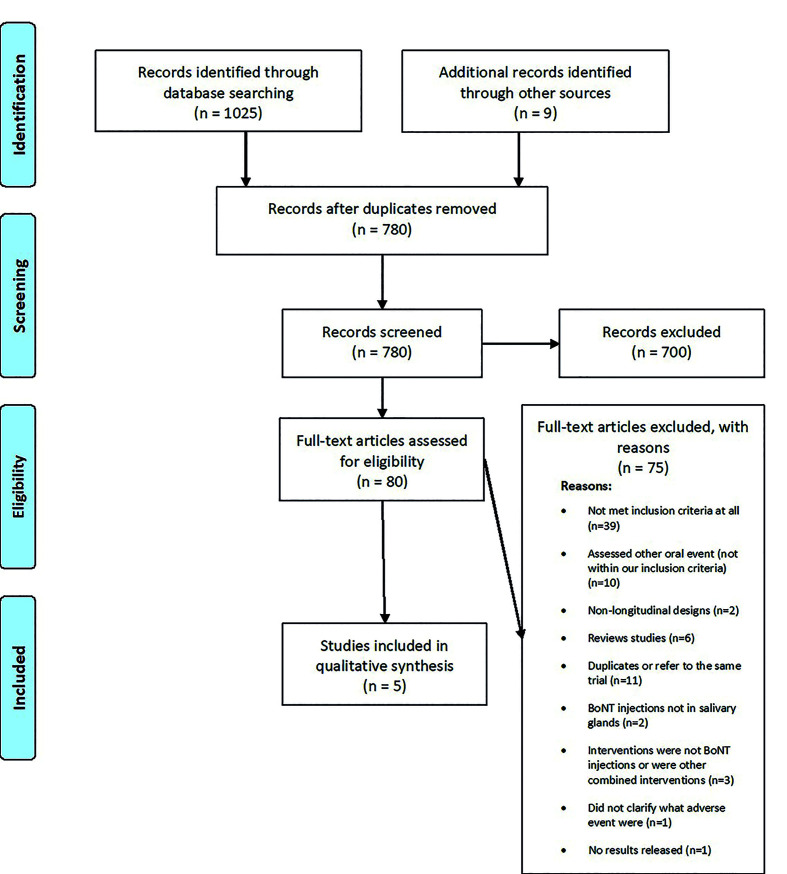
PRISMA flow diagram.

Fig. 1 illustrates the study selection process. We retrieved a total of 1025 records through database searching, and nine through manual search. After removal of duplicates (n=254), 780 studies were screened by title and abstract reading.

Eighty articles were fully read, out of which 5 primary studies were selected and included in this systematic review (2,16,21-23) - of note, among the included studies, information about one of the randomized controlled trials (RCT) (2) was available in conference abstracts, on a poster, on an article supplement, and at clinicaltrials.gov under the identifier NCT01994109.

Two studies included in this review were RCTs (2,21) and three were quasi-experimental studies (16,22,23). The characteristics of included primary studies (along with demographic and clinical characteristics of participants) are presented in Table 1. Out of the five included primary studies, salivary buffering capacity (22,23) was assessed by two; oral pH and *S. mutans* and *Lactobacilli* salivary counts were assessed by three (21-23), and carious lesions were assessed by two (2,16). Interventions and outcomes of included studies are summarized in Table 2.

**Table 1:**
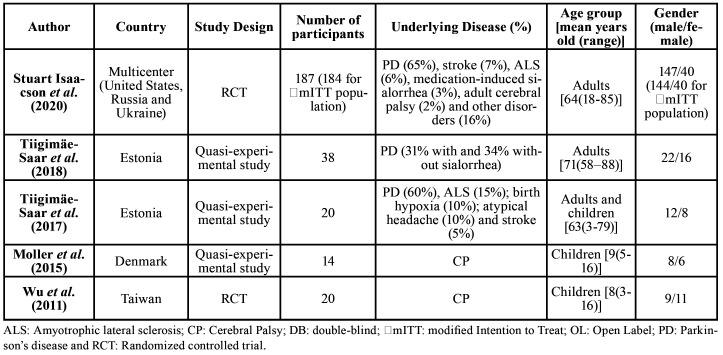
Characteristics, demographics and summary of included studies.

**Table 2:**
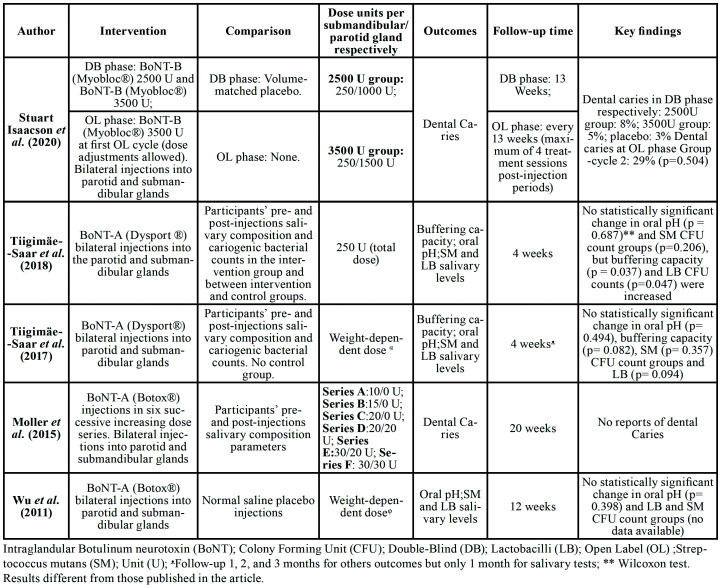
Interventions and outcomes of included studies are summarized.

- Carious lesions

In the double-blind (DB) phase of the multicenter RCT by Stuart Isaacson (2), carious lesions development were observed in 8% of the patients of the 2500 U BoNT group, 5% of the patients of the 3500 U BoNT group, and in 3% of the patients of the placebo group. From these data, we were able to calculate the relative risk of developing new carious lesions (compared to placebo) of 2.4 (95% confidence interval [CI]=0.48-11.80) for the 2500 U group, and 1.4 (95%CI=0.24-8.12) for the 3500 U group. The results of the Chi-square test performed by us did not show statistically significant differences in group comparison (*p*=0.504). Carious lesions were also assessed in the open-label study of Moller *et al*. (16), which reported no cases of tooth decay.

- Salivary buffering capacity

The quasi-experimental studies of Tiigimäe-Saar *et al*. (22,23) found different results concerning the changes in buffering capacity. For the analysis carried out in 2017 (23), no statistically significant change was observed. By contrast, the 2018 study reported significantly increased buffering capacity one month after BoNT injections (22).

- Oral pH

Wu *et al*. (21) did not observe significant differences in participants’ oral pH before and after BoNT-A injections between the active and control groups. There were also no statistically significant differences regarding salivary pH in the two other quasi-experimental studies (22,23) that also analyzed this variable.

- *S. mutans* and *Lactobacilli* salivary counts

The RCT by Wu *et al*. (21) did not provide any data - either in the form of primary data or effect size measures - related to the cariogenic bacterial count presenting only the hypothesis tests results (in the form of *p-value*) for the comparison between the baseline and the post-intervention period. The authors reported no statistically significant changes in *S. mutans* and *Lactobacilli* colony-forming units (CFU) counts. The studies of Tiigimae-Saar did not find statistically significant differences in *S. mutans* CFU counts either(22,23). However, the 2018 study found that *Lactobacilli* CFU counts were significantly increased one month after BoNT injections (22).

- Risk of bias of individual studies

**Figure 2:**
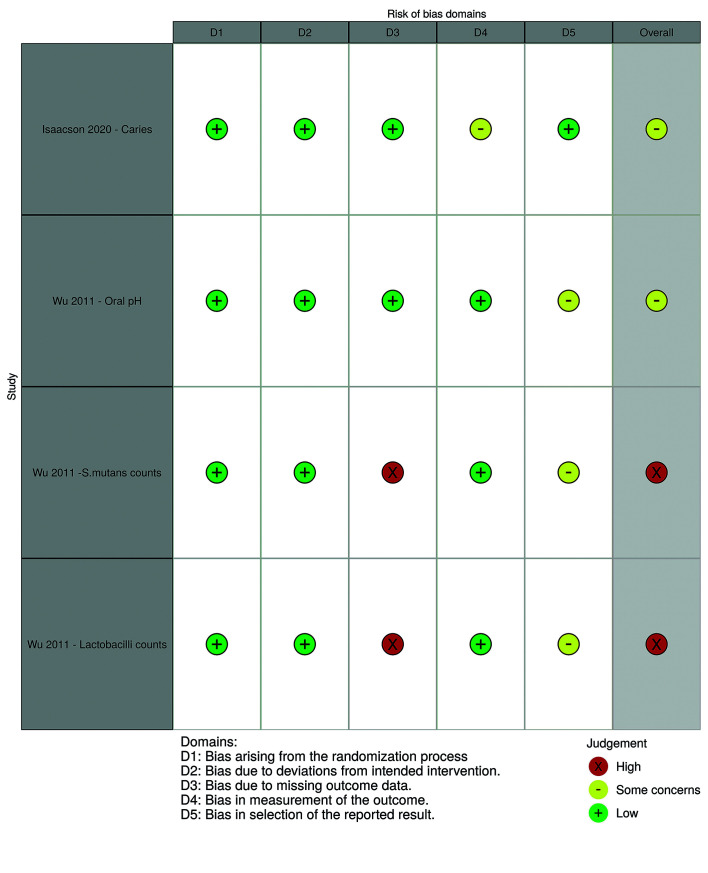
Risk of bias for randomized studies (by robvis tool).

**Figure 3:**
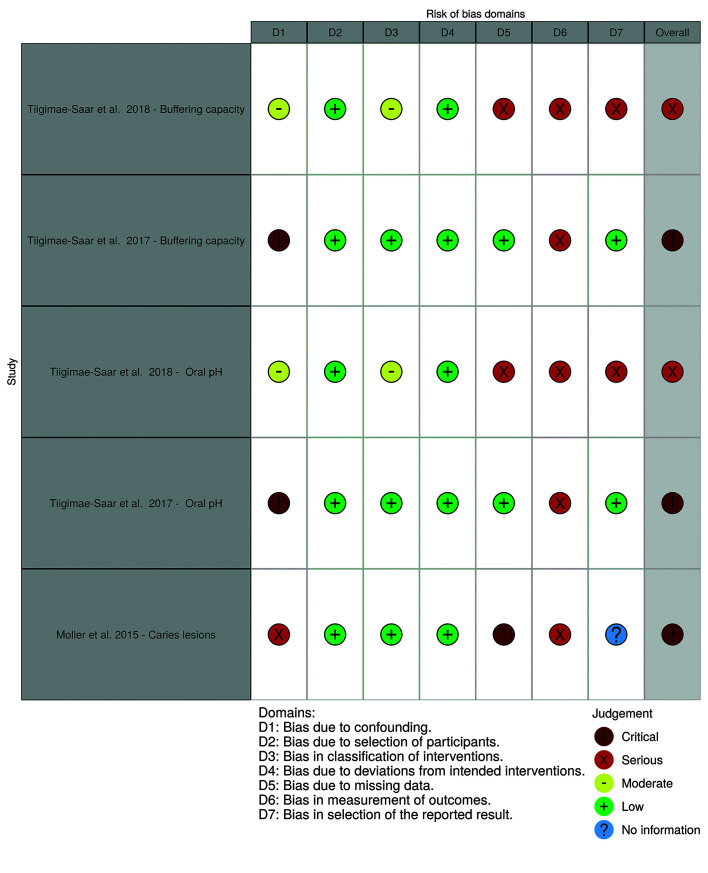
Risk of bias for non-randomized studies for saliva composition and caries (by robvis tool).

**Figure 4:**
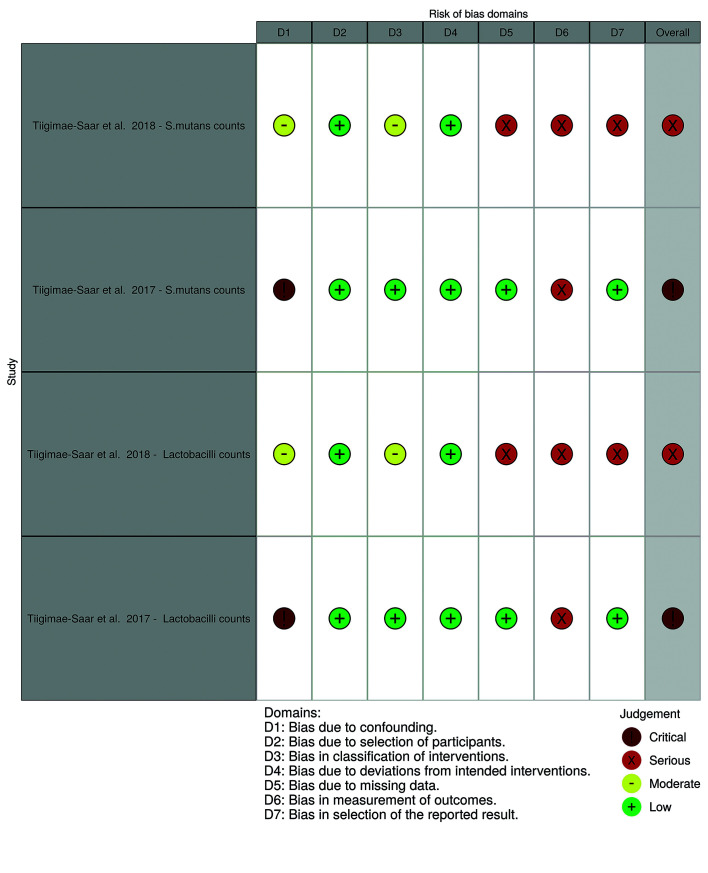
Risk of bias for non-randomized studies for cariogenic bacteria counts (by robvis tool).

Fig. 2 displays the risk of bias classification for randomized studies, while Fig. 3 and Fig. 4 depict such classification for non-randomized studies.

The multicenter randomized trial (2) was overall classified as having “some concerns” on its risk of bias, particularly due to concerns in outcome measurement. This study protocol mentions that four radiographic bitewings were to be taken and that Dental Adverse Event (DAE) criteria were followed as defined in the protocol. However, it is not clear if the independent dentists were calibrated and how the oral examination was performed, how carious lesions were diagnosed, or how were dental caries was defined. Therefore, doubts remain on whether incipient lesions could have been unreported due to the adopted diagnosis methods.

The RCT published by Wu *et al*. (21) was overall judged to be of “some concerns” in relation to oral pH. We have not found any publicly available research protocol or pre-specified analysis plan. Regarding cariogenic bacteria counts, the overall risk of bias was considered high, as no protocol was found and the study only provides the corresponding *p-value*.

As for the non-randomized studies, the study by Moller *et al*. (16) was classified as having a critical risk of bias. Which is explained by the absence of dental caries definition, the possibility of confounding due to the performance of extra oral exams and oral hygiene instructions, and the absence of a control group.

The 2017 study by Tiigimäe-Saar *et al*. (23) was judged to have an overall critical risk of bias for all outcomes. Not only there was no control group, but also there was a serious possibility for confounding. In fact, participants were very heterogeneous regarding their underlying diseases and medication use, with no described approach for adjustment or control of confounding variables. In addition, the outcome measurement involved subjective judgments by the evaluator.

The 2018 study by Tiigimäe-Saar *et al*. (22) was classified as having a serious risk of bias. Although the authors presented results for two control groups, both control groups were not comparable as they did not have hypersalivation (one of the control groups consisted of healthy individuals). There is also a risk of selection bias (namely, indication bias), since only subjects with drooling received BoNT injections. Regarding outcomes measurement, as with the 2017 study (23), subjective measurement methods were used for all outcomes.

## Discussion

Overall, in this systematic review, we did not find any primary study reporting associations between BoNT use and caries development. Regarding the remaining outcomes, only one study found statistically significant differences in salivary composition, particularly in buffer capacity and *Lactobacilli* counts. Therefore, existing evidence suggests that BoNT are safe to use in patients with drooling. However, care should be taken when interpreting these results, as all included studies had at least “some concerns” regarding their risk of bias.

None of the two studies (2,16) assessing carious lesions found BoNT to be associated with caries development. In fact, Moller *et al*. (16) did not report any dental caries, which might be a consequence of the extraoral examinations and hygiene instructions provided during the studied period. Nevertheless, neither of these two studies (2,16) presented the definition used for caries diagnosis. Regarding buffer capacity, despite expectations that the salivary buffer capacity would be reduced and hinder the neutralization process of the oral environment (further reducing the oral pH), Tiigimäe-Saar observed an increased stimulated saliva’s buffer capacity after BoNT injections (22) pointing to saliva’s ability to resist changes in the oral environment balance when challenged. Such differences were not found in the other studies assessing buffering capacity (23) and oral pH (21-23). The differences between studies may be explained by the applied measurement methods (measuring of saliva in different states and lack of measurement concealment in the two (22,23) quasi-experimental studies) and by the intervention characteristics such as dosage used, various types of underlying diseases and inadequate allocation of participants into groups. The Tiigimäe-Saar 2018 study (22) also found a significant increase in *Lactobacilli* counts. *Lactobacilli* are more acidic and a drop in salivary pH may be a factor that favors their increase (24). However, the reduction in the unstimulated saliva’s pH at 2018 study (22) was not statistically significant, leaving doubts about whether the pH reduction contributed to a more accentuated growth of *Lactobacilli*. Other factors that may explain this *Lactobacilli* increase concern (i) the use, in this study (22), of a pre-established dose of BoNT (while the other two studies applied a weight-based dosage), (ii) and differences in information or control of the use of mouthwashes and oral antiseptics (Lactobacilli have bigger resistance to bacteria-reducing substances, such as chlorhexidine, and are more abundant in areas that are difficult to clean (25)).

This systematic review presents some limitations, mostly related to the included primary studies. Firstly, there was limited evidence for some outcomes - including caries risk and salivary osmolarity -, which were not assessed by all primary studies. In addition, the methods of outcomes assessment may also be a matter of concern - (i) pH, buffer capacity and bacterial counts were subjectively assessed in both Tiigimäe-Saar studies (22,23), and (ii) it is not clear whether saliva samples are appropriate for evaluating the microbiology of oral diseases. In this respect, the literature is controversial, with some studies claiming that such samples are appropriate (26,27) and others not (28,29). There are also important concerns related to primary studies’ sample sizes. In fact, only the multicenter RCT (2) mentioned having performed a sample size calculation. Therefore, the included studies were probably underpowered to detect relevant differences for all endpoints of this review.

Lack of control for confounding factors should also be taken into account. One of such factors concerns participants’ underlying diseases. In Parkinson’ disease (PD), previous studies have found drooling to be more frequent in patients of older age, with more severe presentations (30), and with longer disease duration (22). The amount of saliva produced also appears to mirror the leading PD symptom (22), being akinesia-rigidity the most frequent disease subtype among PD patients with sialorrhea, and tremor the most frequent subtype among PD patients without sialorrhea (22). On the other hand, in patients with cerebral palsy, their neuromotor abnormality type may also affect the salivary parameters - patients with spastic palsy appear to have lower saliva flow rate, as well as lower pH and buffer capacity (31). Another key confounding factor concerns the potential influence of medication. The use of medications that interfere with drooling was not allowed in some of the studies (16,22,23) that were included in this systematic review. However, the effects that many drugs have on saliva may not yet be known. As the multicenter trial was the only study in which randomization was performed (2), this is the only study for which samples may be comparable regarding medication use.

Regarding BoNT injection sites and doses, the studies varied widely, which may have influenced the observed results as well. For example, as parotid secretions predominate in the stimulated state (32) and produce a watery saliva (33,34), higher BoNT injections doses into those glands could, along with increased salivary osmolality and reduced buffering capacity, reduce the removal of food debris (as there would be lower water content in saliva) and increase caries risk. On the other hand, submandibular gland secretions predominate in the in the unstimulated state (32) with a lower concentration of bicarbonate (35) and, when salivary flow is lower, acid by-products may remain in longer contact with oral structures (32) and increasing caries risk.

Finally, we did not analyze all oral changes that can potentially result from BoNT treatment. For example, we were not able to assess the effect of BoNT in the salivary concentration of proteins and electrolytes, or in the counts of bacteria other than *S. mutans* and *Lactobacilli* (the latter issue can be particularly relevant as dental caries is a dysbiosis, with *S. mutans* and *Lactobacilli* representing a small percentage of mouth bacteria (28)).

Despite these limitations, this systematic review has important strengths. In order to minimize the risk of selection bias and the impact of publication bias, we used a comprehensive query, and searched in four bibliographic databases, with electronic searches being complemented by manual search methods. Primary studies’ selection and quality assessment was performed according to Cochrane recommended practices. Following data extraction, we contacted the authors of primary studies to obtain missing information. Finally, to the best of our knowledge, this is the first systematic review on the topic, synthesizing and evaluating the quality of the existing evidence.

In conclusion, this systematic review has not found evidence that, in patients with drooling, BoNT injections associate with caries development or disturbances in oral pH, buffer capacity and cariogenic bacterial counts. However, we cannot yet affirm that BoNT is completely safe, as included primary studies had important limitations and differences in their methodologies. Therefore, future studies - preferentially RCT - should be conducted, adopting standardized procedures and adequately controlling for confounding. On Supplement1 (http://www.medicinaoral.com/medoralfree01/aop/24101_Supplement1.pdf), we list a series of clinical and methodological recommendations for future studies assessing the effect of BoNT in patients with drooling.
